# Two-Step In Vitro Model to Evaluate the Cellular Immune Response to SARS-CoV-2

**DOI:** 10.3390/cells10092206

**Published:** 2021-08-26

**Authors:** Juliana G. Melgaço, Tamiris Azamor, Andréa M. V. Silva, José Henrique R. Linhares, Tiago P. dos Santos, Ygara S. Mendes, Sheila M. B. de Lima, Camilla Bayma Fernandes, Jane da Silva, Alessandro F. de Souza, Luciana N. Tubarão, Danielle Brito e Cunha, Tamires B. S. Pereira, Catarina E. L. Menezes, Milene D. Miranda, Aline R. Matos, Braulia C. Caetano, Jéssica S. C. C. Martins, Thyago L. Calvo, Natalia F. Rodrigues, Carolina Q. Sacramento, Marilda M. Siqueira, Milton O. Moraes, Sotiris Missailidis, Patrícia C. C. Neves, Ana Paula D. Ano Bom

**Affiliations:** 1 Instituto de Tecnologia em Imunobiológicos, Bio-Manguinhos, Fundação Oswaldo Cruz, FIOCRUZ, Rio de Janeiro 21040-900, Brazil; tamiris.azamor@bio.fiocruz.br (T.A.); amarques@bio.fiocruz.br (A.M.V.S.); jose.henrique@bio.fiocruz.br (J.H.R.L.); tiago.pereira@bio.fiocruz.br (T.P.d.S.); ygara.mendes@bio.fiocruz.br (Y.S.M.); smaria@bio.fiocruz.br (S.M.B.d.L.); camilla.bayma@bio.fiocruz.br (C.B.F.); jane.silva@bio.fiocruz.br (J.d.S.); fonseca@bio.fiocruz.br (A.F.d.S.); luciana.tubarao@bio.fiocruz.br (L.N.T.); danielle.cunha@bio.fiocruz.br (D.B.e.C.); tbomfimsantos@gmail.com (T.B.S.P.); menezescat@outlook.com (C.E.L.M.); sotiris.missailidis@bio.fiocruz.br (S.M.); Pcristina@bio.fiocruz.br (P.C.C.N.); adinis@bio.fiocruz.br (A.P.D.A.B.); 2 Laboratório de Vírus Respiratório e do Sarampo, Instituto Oswaldo Cruz, Fundação Oswaldo Cruz, FIOCRUZ, Rio de Janeiro 21040-900, Brazil; milene_dias@yahoo.com.br (M.D.M.); alinematos@hotmail.com (A.R.M.); braulia.costa@gmail.com (B.C.C.); jessicadl_10@hotmail.com (J.S.C.C.M.); mmsiq@ioc.fiocruz.br (M.M.S.); 3 Laboratório de Hanseníase, Instituto Oswaldo Cruz, Fundação Oswaldo Cruz, FIOCRUZ, Rio de Janeiro 21040-900, Brazil; thyagoleal@yahoo.com (T.L.C.); milton.moraes@fiocruz.br (M.O.M.); 4 Laboratório de Imunofarmacologia, Instituto Oswaldo Cruz, Fundação Oswaldo Cruz, FIOCRUZ, Rio de Janeiro 21040-900, Brazil; nataliafintelman@gmail.com (N.F.R.); carol.qsacramento@gmail.com (C.Q.S.); 5 Centro de Desenvolvimento Tecnológico em Saúde, Fundação Oswaldo Cruz, FIOCRUZ, Rio de Janeiro 21040-900, Brazil

**Keywords:** in vitro model, immunity, SARS-CoV-2

## Abstract

The cellular immune response plays an important role in COVID-19, caused by SARS-CoV-2. This feature makes use of in vitro models’ useful tools to evaluate vaccines and biopharmaceutical effects. Here, we developed a two-step model to evaluate the cellular immune response after SARS-CoV-2 infection-induced or spike protein stimulation in peripheral blood mononuclear cells (PBMC) from both unexposed and COVID-19 (primo-infected) individuals (Step1). Moreover, the supernatants of these cultures were used to evaluate its effects on lung cell lines (A549) (Step2). When PBMC from the unexposed were infected by SARS-CoV-2, cytotoxic natural killer and nonclassical monocytes expressing inflammatory cytokines genes were raised. The supernatant of these cells can induce apoptosis of A549 cells (mock vs. Step2 [mean]: 6.4% × 17.7%). Meanwhile, PBMCs from primo-infected presented their memory CD4^+^ T cells activated with a high production of *IFNG* and antiviral genes. Supernatant from past COVID-19 subjects contributed to reduce apoptosis (mock vs. Step2 [ratio]: 7.2 × 1.4) and to elevate the antiviral activity (iNOS) of A549 cells (mock vs. Step2 [mean]: 31.5% × 55.7%). Our findings showed features of immune primary cells and lung cell lines response after SARS-CoV-2 or spike protein stimulation that can be used as an in vitro model to study the immunity effects after SARS-CoV-2 antigen exposure.

## 1. Introduction

The infection caused by the new coronavirus (SARS-CoV-2) induces a severe acute respiratory syndrome, with signals and symptoms such as fever, cough, and pneumonia, namely COVID-19 [1,2]. This virus of high transmissibility and high mortality rates among respiratory infections has collapsed the intensive care units of hospitals around the world [1,2]. The only preventive measure to control the spread of this disease is vaccination combined with reducing social activities, wearing adequate masks, and hand sanitization [3,4,5,6,7]. As the SARS-CoV-2 variants were emerging in some parts of the globe, the pandemic showed its strength scaling up new cases and mortality rates, thus contributing to several countries joining in a race for mass immunization [3,4,5,6,7].

SARS-CoV-2 is a single-stranded, positive-stranded RNA virus between 60 and 140 nm in diameter with corona-like projections on its surface, formed by the spike glycoproteins (protein S), crucial for invading target cells [8]. Immune response is a key part of the clinical evolution of the disease, in which cytokine storm, leucopenia, and lung infiltrating macrophages and neutrophils are associated with poorer outcomes [2,9,10]. Nevertheless, some cellular immune responses during COVID-19 were described as crucial for host defense [2,11], where the main points are the heterogeneity of the immune system and potentially distinct patient immunotypes [12]. However, few studies are focused on Latin American countries, in which socio-economic issues are always challenging and genetic factors can influence different responses [13].

Viral reactivation and reinfection have been described and associated with successive contact with different SARS-CoV-2 strains [14,15,16]. In this way, memory phenotypes raised after infection should be more explored and compared with the phenotypes activated after vaccination [17,18,19,20,21]. Therefore, the function of immune response cells under live virus exposition is not well understood even in exposed or unexposed individuals to SARS-CoV-2 [22], and how the product of this cellular response can influence the pulmonary alveolar epithelial cells is not fully understood. Here, we developed an in vitro model to evaluate the events induced by live SARS-CoV-2 and spike protein in different subsets of immune in vitro cells (Step1), as well as the effects of these cellular immune response products in a lung cell line in the presence of SARS-CoV-2 (Step2). This two-step in vitro model provides useful information for a better understanding of the host interactions with SARS-CoV-2 and could guide future evaluation of vaccines and biopharmaceuticals.

## 2. Materials and Methods

### 2.1. Study Design

In this study, the goal is to present a two-step model to evaluate the cellular immune response against SARS-CoV-2 (whole virus) using in vitro cell cultivation carried out in a biosafety level III laboratory. The study was designed with the aim of investigating the cellular immune response using peripheral blood mononuclear cells (PBMC) from volunteers by antigenic stimulation, with spike protein and virus, as the first step (Step1). After that, the harvested supernatant from the immune cells was used in the cultivation of lung alveolar epithelial cells to evaluate antiviral/pathological effects of the cellular immune response, as the second step (Step2).

PBMC from naïve healthy volunteers and primo-infected COVID-19 individuals (without hospitalization), with one previous detection of SARS-CoV-2 by naso-oropharyngeal swabs RT-qPCR were submitted to cellular cultivation assay in the presence of live SARS-CoV-2 (infection-induced) and/or spike protein (antigen stimulation) [23]. PBMC were incubated for 48 h at 37 °C, in a humidified chamber, at 5% CO_2_ and maintained with RPMI-1640 media as negative control (mock). Naive healthy volunteers are called unexposed from now on, and primo-infected COVID-19 individuals are called COVID-19 from now on (Table 1 and Table 2).

The protocol was performed with virus-infected or spike protein-stimulated PBMC (multiplicity of infection (MOI): 0.01 or 10 µg/mL for spike protein), as a control for viral antigen. Cells were tested by flow cytometry and gene expression (Step1). Human adenocarcinoma alveolar basal epithelial cells (A549-CCL-185) were cultured without and with the virus (MOI: 0.1), as well as with the resultant PBMC supernatants from Step1 to assess the effects of the soluble mediators from the cellular immune response in lung alveolar epithelial cells (Step2). All experiments were performed independently and in triplicates.

### 2.2. Samples

Blood samples were obtained from volunteers (*n* = 29, mean ± SE = 36 ± 1.9 years old, BMI < 30, 13 males) without SARS-CoV-2 infection at intake (RT-qPCR negative) (Table 1). Fifteen subjects showed no COVID-19 signals or symptoms and were negative for IgM/IgG SARS-CoV-2 antibodies. Three frozen samples were collected before the COVID-19 outbreak (June, July, and November 2019). Eleven subjects tested positive for SARS-CoV-2 infection by RT-qPCR according to the protocol described by Matos et al., 2020 [24] or IgM/IgG antibodies (>two–three months before blood collection), and all presented signals or symptoms of COVID-19. All volunteers were monitored weekly since March 2020, by RT-qPCR on naso-oropharyngeal swabs for SARS-CoV-2 detection [24] (Table 2).

SARS-CoV-2 strain was provided by Laboratório de Vírus Respiratório e do Sarampo, Instituto Oswaldo Cruz, Fiocruz (https://nextstrain.org/ncov: Brazil/RJ-314/2020 or GISAID #414045). It was not possible to confirm if the viral strain used in the experiments was the same that affected the volunteers, but in the meantime, six different strains of SARS-CoV-2 circulating in Brazil were reported [25]. Spike protein was provided by Laboratório de Engenharia de Cultivos Celulares (Lecc), Coppe/UFRJ. Cultivation assays using live virus were performed in a Biosafety Level III laboratory at FIOCRUZ. After viral inactivation using commercial reagents (from RNeasy Plus mini kit, Qiagen and Cytofix, BD Biosciences, San Diego, CA, USA), the final procedures of samples analysis and other experiments with cell cultivation were carried out in a Biosafety Level II laboratory at FIOCRUZ.

#### Ethical Statement

All procedures were performed according to the Helsinki Declaration for ethical procedures with registration number CAAE 34728920.4.0000.5262. All participants provided written informed consent (IRB# 34728920.4.0000.5262).

### 2.3. Laboratory Assays

#### 2.3.1. Viral Detection by RT-qPCR

After 48 h of cell cultivation, 1 × 10^6^ of live PBMC were harvested and centrifuged (400× *g*, 5 min, 4 °C) in the presence or absence of the virus. PBMC and supernatant samples were used to extract viral RNA using RNeasy Plus Mini Kit (Qiagen) and submitted to RT-qPCR assay (Molecular Kit SARS-CoV-2 (E/RP), Bio-Manguinhos, Rio de Janeiro, Brazil) to detect SARS-CoV-2 genome as described by Matos et al., 2020 [24].

#### 2.3.2. Step1: In Vitro Antigen-Specific Cellular Immune Response

To evaluate different subpopulations after infection, PBMC were harvested and washed in FACS buffer solution (2% fetal bovine serum in phosphate buffer solution, pH = 7.4). The cells were centrifuged at 400× *g* for 10 min; the supernatant was collected and frozen at −70 °C. The cells’ pellet was homogenized and then stained with live/dead cell viability dye (Thermo Fisher Scientific, L23105, Waltham, MA, USA) according to the manufacturer’s protocol. The cells were washed in FACS buffer and stained with surface antibodies CD3-APC-Cy7, clone: SP34; CD4-PE, clone: RPA-T4; CD8-Brilliant Violet 605, clone: SK1; CD14-Brilliant Blue 700, clone: MOP9; CD16-FITC, clone: 3G8; CD56-PE-Cy5, clone: B159; HLA-DR-Brilliant Violet 650, clone: G46-6; CD69-Brilliant Violet 421, clone: FN50; CD95-PE-Cy7, clone: DX2; CD45RA-APC, clone: HI100; CCR7 (CD197) Brilliant Violet 510, clone: 3D12; CD19-PE-Cy5, clone: HD37. All from BD Biosciences, San Diego, CA, USA. After surface staining, cells were washed with FACS buffer and fixed (Cytofix, BD Biosciences, USA). To assess SARS-CoV-2 replication on PBMC, the antibodies to dsRNA were used (mouse monoclonal antibody (MAb) J2 Scion, Budapest, Hungary) [26]. The secondary antibody used to detect J2 (Mab) was chicken antimouse Alexa Fluor 488 (1:1000) purchased from Invitrogen (cat#A-21200, Waltham, MA, USA).

Intracellular staining (ICS) assay was also performed for cytokines detection to define subpopulations of CD4^+^ and CD8^+^ T cells after 48 h with spike protein stimulation. Stimulation was performed using 10 μL/mL anti-CD28/49d (BD Biosciences) and 10 μg/mL of spike protein (except for the unstimulated condition) in RPMI1640 medium at a final volume of 500 μL. Furthermore, 1× cell stimulation cocktail of PMA and ionomycin was used as a positive control (eBioscience). Cells were incubated for 48 h at 37 °C, and 1× protein transport inhibitor (eBioscience) was added in the last 4 h of incubation. At the end of incubation time, cells were washed and stained using a viability dye (Live/Dead Fixable Blue; Thermo Fisher Scientific) according to the manufacturer’s protocol. Staining was carried out using the following surface antibodies as described above CD3-APC-Cy7, clone: SP34; CD4-PE, clone: RPA-T4; CD8-Brilliant Violet 605, clone: SK1; HLA-DR-Brilliant Violet 650, clone: G46-6; CD69-Brilliant Violet 421, clone: FN50; CD45RA-APC, clone: HI100; CCR7 (CD197) PE, clone: 3D12. After surface antigen staining, cells were washed twice in FACS buffer, and a fixation–permeabilization step was performed using the FOXP3 Transcription Factor Staining buffer set, according to the manufacturer’s protocol (Ebioscience kit, cat#00-5523-00). The following antibodies were used for intracellular staining (IFN-γ BV510, clone: B27; IL-2 Brilliant Violet 421, clone: MQ1-17H12; IL-4 Brilliant Violet 711, clone: MP4-25D2; TNF PE-Cy7, clone: MAB11). All antibodies were purchased from BD Biosciences. Compensation beads (UltraCompeBeads, Invitrogen^TM^, Carlsbad, CA, USA cat#01-2222-42; ArC^TM^ Armine Reactive Compensation Bead kit, Invitrogen^TM^, Carlsbad, CA, USA, cat#A10346) were used for compensation set-up. The unstimulated condition was used to subtract any background staining off the analysis. Positive ICS or after spike protein stimulation or virus infection was determined by detection of at least 10 SARS-CoV-2-specific T cells and a frequency of SARS-CoV-2-specific T cells of at least twice the corresponding unstimulated signal. Twenty-nine subjects were used for spike protein assay (*n* = 11 COVID-19 and *n* = 18 unexposed), and sixteen were enrolled for live virus assay (*n* = 6 COVID-19 and *n* = 10 unexposed) (Table 1).

**Figure 1:**
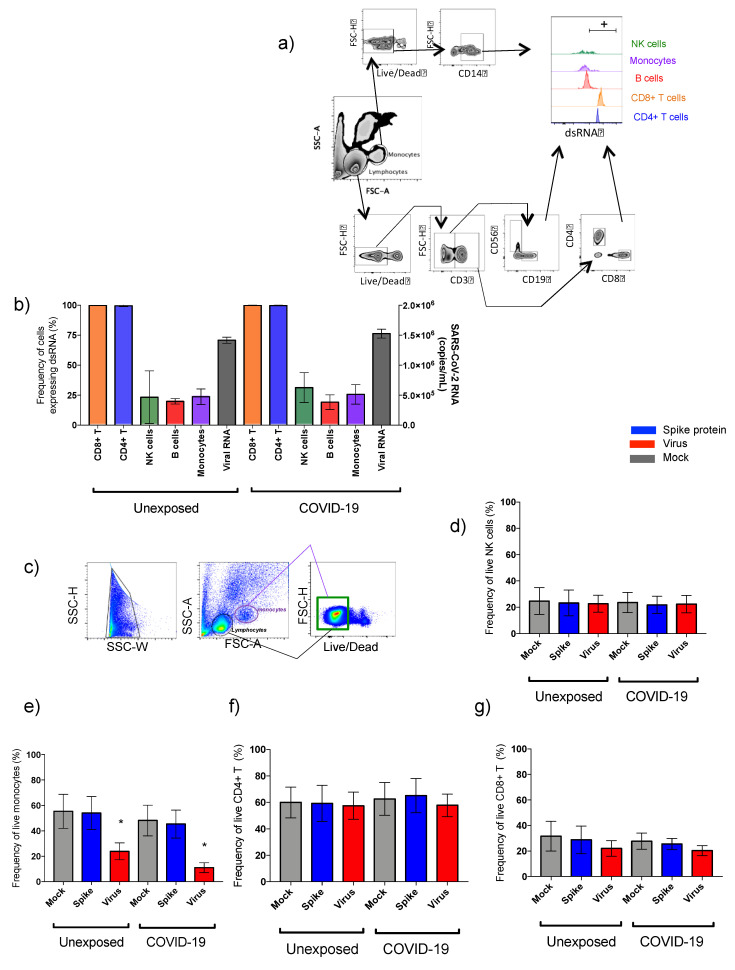
Presence of SARS-CoV-2 in PBMC and the percentage of live phenotypes exposed to spike protein and to live SARS-CoV-2. Gate strategy to get the subpopulations evaluated. (**a**). Viral load of RNA detected on supernatant from unexposed and COVID-19 PBMC cultured with live virus and frequency of double-strand (ds) RNA detection in different phenotypes, as CD8^+^T, CD4^+^T, natural killer (NK), B lymphocytes, and monocytes after in vitro cultivation (**b**). Gate to define total live PBMCs are represented with green lines (FSC-H vs. Live/Dead) with lymphocytes in black and monocytes in purple on the backgating (**c**). Subsequently, to separate natural killer from CD4^+^ and CD8^+^ T cells, we used CD56 marker to natural killer, and CD3 marker to define the T-cells subpopulation. Data from PBMC cultured with spike protein (blue) and SARS-CoV-2 (red) on total live natural killer (NK: CD56^+^CD3^−^) (**d**), total live monocytes (**e**), and total live T lymphocytes (CD3^+^CD4^+^) (**f**) and (CD3^+^CD8^+^) (**g**). The data are representative of 2–5 different experiments. * *p* < 0.05, ** *p* < 0.01, and *** *p* < 0.001.

**Figure 2:**
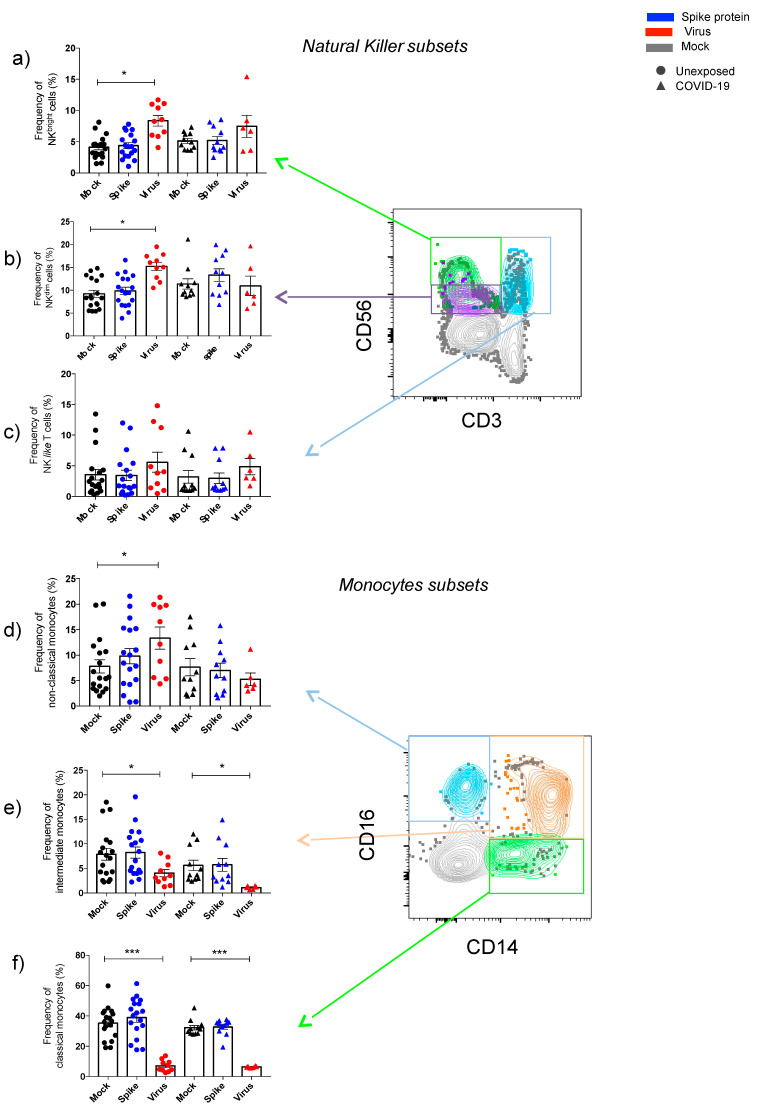
Effects of SARS-CoV-2 on natural killer and monocytes subsets. The percentage of natural killer (NK) subsets are displayed for NK^bright^ (CD3^−^CD56^high^CD16^low^) (**a**: green color in the dot plot), NK^dim^ (CD3^−^CD56^low^CD16^high^) (**b**: purple), and NK-like T cells (CD3^+^CD56^+^) (**c**: blue) in both groups, unexposed and SARS-CoV-2 exposed (COVID-19), up to 48 h of cell cultivation in the presence of spike protein and live SARS-CoV-2. The percentage for monocytes subsets were divided in nonclassical monocytes (CD14^−^CD16^+^) (**d**: blue color), intermediate monocytes (CD14^+^CD16^+^) (**e**: orange color), and classical monocytes (CD14^++^CD16^−^) (**f**: green) on cell cultivation with antigens. The data are representative of 2–5 different experiments. * *p* < 0.05, ** *p* < 0.01, *** *p* < 0.001.

**Figure 3:**
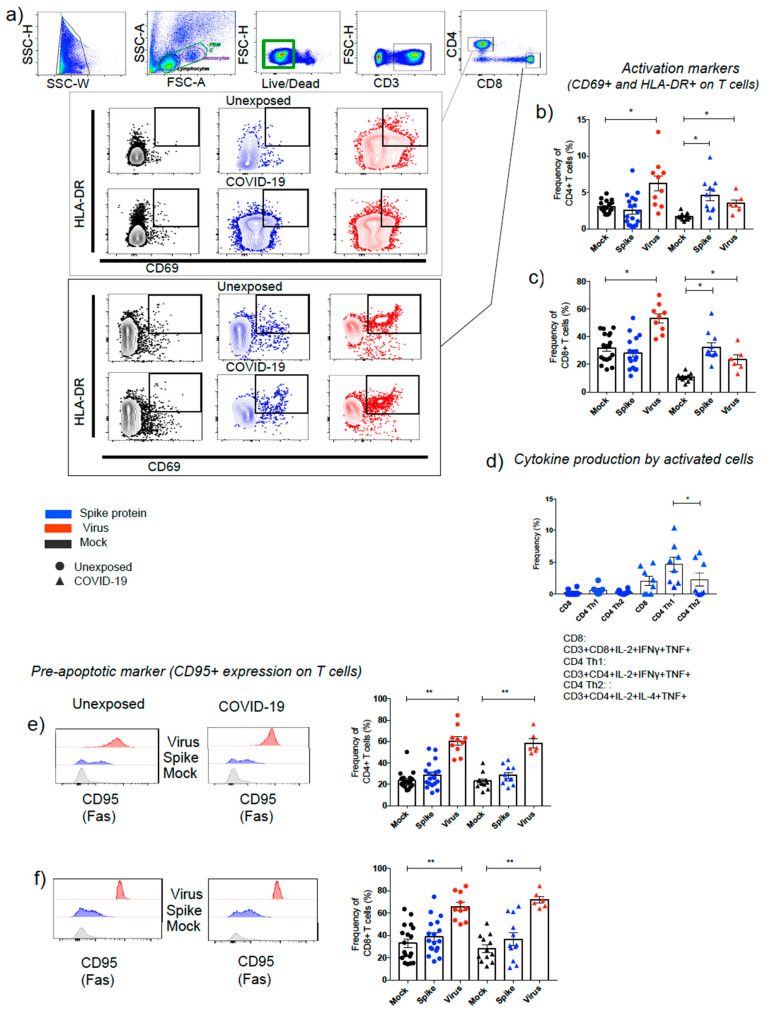
SARS-CoV-2 effects on lymphocytes T activation and pre-apoptosis. Gate strategy to define the subpopulations using dot plot FSC vs. SSC (green dashed line represents PBMC, purple line is the monocytes, and black line is the representation of lymphocytes. Gate to define total live PBMCs are represented with green line (FSC-H vs. Live/Dead). After gating live CD3^+^ T cells, it was possible to set activated T cells (CD4^+^ and CD8^+^), showing nonstimulated (black), stimulated with spike protein from SARS-CoV-2 (blue), and exposed with live virus (red) (**a**). Percentages obtained from unexposed and COVID-19 displaying data for CD4^+^ (**b**) and CD8^+^T cells (**c**) activated by antigens. The intracellular staining assays with spike protein is showing CD8^+^ T lymphocytes producing IL-2, TNF and IFN-gamma, as well as CD4^+^ subsets, CD4^+^ Th1 and CD4^+^ Th2 with their main representative cytokines production by those cells in both groups, unexposed and SARS-CoV-2 exposed (COVID-19) (**d**). The expression of pre-apoptotic marker (CD95^+^) on CD4^+^ (**e**) and on CD8^+^ T cells (**f**) is displayed. The data are representative of 2–5 different experiments. * *p* < 0.05, ** *p* < 0.01, and *** *p* < 0.001.

All the steps were performed at 4 °C unless otherwise specified by the manufacturer. The acquisition was performed using LSR Fortessa flow cytometer (Becton Dickinson, San Diego, CA, USA), and data were analyzed using FlowJo^TM^ software (Becton Dickinson). Gate strategy to analyze immune cells subpopulations and alveolar epithelial cells by flow cytometry is summarized in Figures 1–3, 5 and 7.

#### 2.3.3. Cytokines/Chemokines Gene Expression

To evaluate the gene expression profile after challenge, same cellular RNA extracted for viral detection was quantified in spectrophotometer (Nanodrop Technologies, Waltham, MA, USA), followed by a complementary DNA (cDNA) synthesis from 250 ng of total RNA using the High-Capacity cDNA Reverse-Transcription Kit (Thermo Fisher Scientific), both procedures according to manufacturer’s instructions. Analysis of gene expression was performed using Fluidigm (Biomark platform) assays, as described elsewhere [27].

Briefly, the software R Studio v. 3.4.0 custom scripts were used for parsing raw foreground and background intensities, exported from the commercial Fluidigm^®^ software. Fluorescence accumulation and melting curve graphs of Rn for each reaction with each gene were inspected. For quantification of relative gene expression, the fluorescence accumulation data of each sample were used for fitting four-parameter sigmoid curves using the ‘qpcR’ v. 1.4.1 R package. For each gene, efficiency was estimated by the mean of all efficiencies. Reference genes *18S*, *GAPDH* and *RPL13* were chosen based on stability measure from geNorm method. Sample-wise normalization factors were calculated by taking the geometric average of selected reference genes [28,29].

#### 2.3.4. Step2: Human Alveolar Lung Epithelial Cells (A549) Cultivation with Supernatant from PBMC Cultured with SARS-CoV-2

Infected (MOI:0.1) and noninfected A549 cells (ATCC-CCL-185) 5 × 10^5^/well were cultured in a 24-well plate for 48 h at 37 °C, in a humidified chamber, at 5% CO_2_ with a pool of defrost supernatant from PBMC cultivated with live SARS-CoV-2 particles, from Step1. A549 cells were harvested to evaluate the expression of nitric oxide synthase (iNOS) (iNOS/NOS2-FITC, cat#610331, BD Biosciences) and caspase-3 (VP-C308, Vector Labs) expression in lung cells by flow cytometry. The dsRNA staining was performed to detect the percentage of virally infected A549 cells, as previously described for PBMC samples (item 2.3.2). The secondary antibody used to detect caspase-3 and dsRNA was chicken antimouse Alexa Fluor 647 (1:1000) purchased from Invitrogen (cat#A-21463, Waltham, MA, USA). The viral infection on A549 cells was performed to distinguish the effects induced by the virus and/or by the products of cellular immune responses.

### 2.4. Statistical Analysis

The normality assumption of the data was initially evaluated by the Kolmogorov–Smirnov test or Shapiro–Wilk. Differences between groups were assessed using the Mann–Whitney *t* test, considering the investigation groups. Bar graphs are represented as mean ± standard error (SE). The software GraphPad Prism for Macintosh, version 8.4.2 (San Diego, CA, USA) was used to perform statistical analysis. The significance for all statistical analyses was defined as *p* < 0.05.

Baseline signals of activated cells in unstimulated controls were subtracted from SARS-CoV-2-stimulated assays to enable the visualization of SARS-CoV-2-induced or SARS-CoV-2-specific cell signals. Cell frequencies above one percent were considered for the final analysis.

## 3. Results

### 3.1. SARS-CoV-2 Replication on Mononuclear Cells Subsets

The first analysis was aimed at investigating the presence of SARS-CoV-2 replication inside the peripheral blood mononuclear cells. All blood samples were negative for SARS-CoV-2 RNA by qPCR before infection of the cell culture. The replicative dsRNA as well as significative viral loads in supernatants were detected after 48 h of virus exposure of PBMCs from all individuals (Figure 1b).

Overall, the analysis of absolute frequency of phenotypes before stimulation showed that lymphocytes (T and B cells) were more frequent than monocytes and natural killer cells in the peripheral blood cells from all participants, showing no difference between unexposed and COVID-19 groups (Table 3).

The viral replication (dsRNA) was found in all cellular phenotypes explored, as monocytes, lymphocytes T and B, and natural killer cells. Our data show that viral SARS-CoV-2 infectivity in PBMC was similar for COVID-19 and unexposed subjects (Figure 1a,b).

### 3.2. Step1: SARS-CoV-2 Effects on the Functionality of PBMC Phenotypes and Cytokine Gene Expression

No changes in live cells percentages using PBMC from unexposed and COVID-19 were detected after in vitro spike protein exposure (Figure 1). However, a strong reduction of total live monocytes was observed (Figure 1e) in the evaluation of phenotypes after virus exposition (mock vs. virus (mean): unexposed: 55.4% × 23.9%, *p* < 0.0001; COVID-19: 48.2% × 11.0%, *p* < 0.0001).

#### 3.2.1. Natural Killer Cells

**Figure 4:**
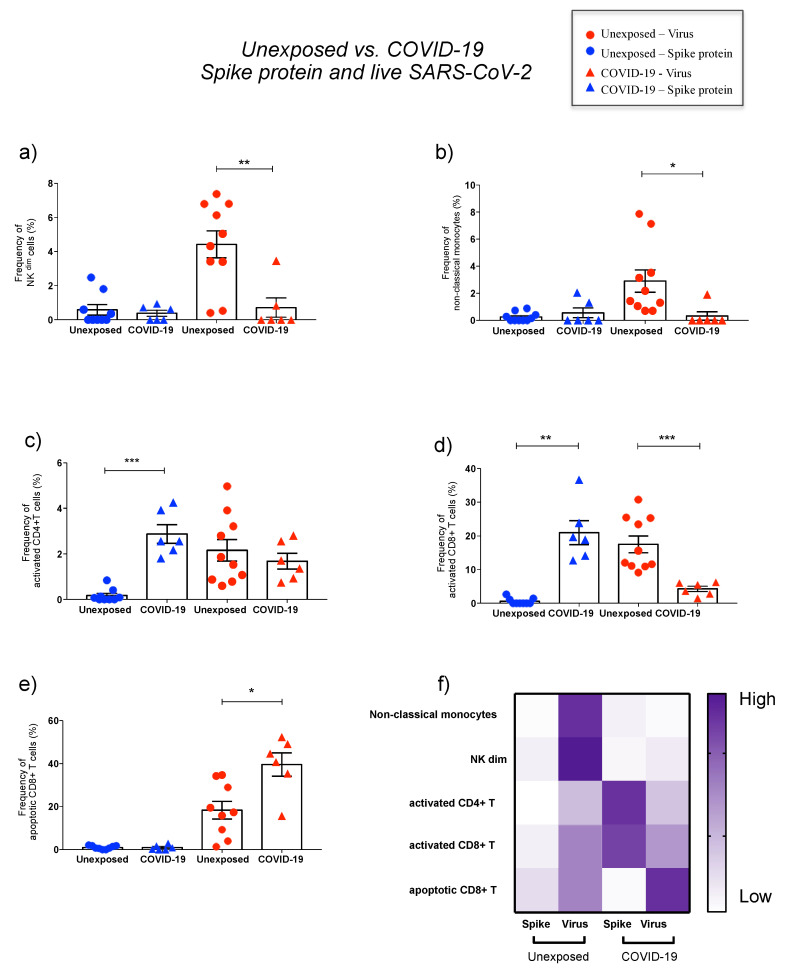
Major effects of spike protein and live SARS-CoV-2 on PBMC phenotypes comparing unexposed and COVID-19 groups. Percentage of natural killer cytotoxic subset (NK^dim^, CD3^−^CD56^low^CD16^high^) (**a**) and nonclassical monocytes (CD3^−^CD14^−^CD16^+^) (**b**) in unexposed (circles) and COVID-19 (triangles) after spike protein (blue) and in vitro virus exposure (red). Percentage of activated (CD69^+^HLA−DR^+^) CD4^+^ (**c**) and CD8^+^T lymphocytes (**d**), as well as apoptotic (CD95^+^) CD8^+^ T lymphocytes (**e**) after antigen stimulation (spike protein and virus). Heat map of phenotypes percentages affected by spike protein and live virus stimulation on unexposed and COVID-19 (**f**). The final value percentage represented by *y* axis is the percentage of cells activated by SARS-CoV-2 antigens subtracted from unstimulated cells (% of cells stimulated with spike protein or virus minus % of the cells from mock condition). Symbols legend represents each sample collected from each volunteer. The data are representative of 2–5 different experiments. * *p* < 0.05, ** *p* < 0.01, and *** *p* < 0.001.

Regarding subsets of SARS-CoV-2-infected natural killer cells, significant differences were documented for unexposed and COVID-19 (Figure 2a–c and Figure 4a,f). Natural killer subsets (NK^bright^ [CD56^high^CD16^low^CD3^−^] and NK^dim^ [CD56^low^CD16^high^CD3^−^]) showed a significant increase in their percentage after virus infection in unexposed individuals, while the same result was not observed for COVID-19 (Figure 2a–c). In addition, NK^dim^ (CD56^low^CD16^high^CD3^−^) frequencies were significantly higher in unexposed than COVID-19 after contact with live SARS-CoV-2 (Figure 4a,f). In contrast, after spike protein stimulation, no differences in NK subsets percentages were observed (Figure 2a–c and Figure 4a,f).

#### 3.2.2. Monocytes

Classical (CD14^+^CD16^−^) and intermediate (CD14^+^CD16^+^) monocytes percentages decreased significantly after in vitro SARS-CoV-2 infection-induced in all subjects (Figure 2d–f). Unexposed subjects presented a significant increase (Figure 2d–f), and the highest percentages of nonclassical monocytes compared to COVID-19 (Figure 4b,f). In contrast, no changes were observed in monocytes subsets percentages after spike protein stimulation (Figure 2d–f and Figure 4b,f).

#### 3.2.3. CD4^+^ T and CD8^+^ T Lymphocytes

**Figure 5:**
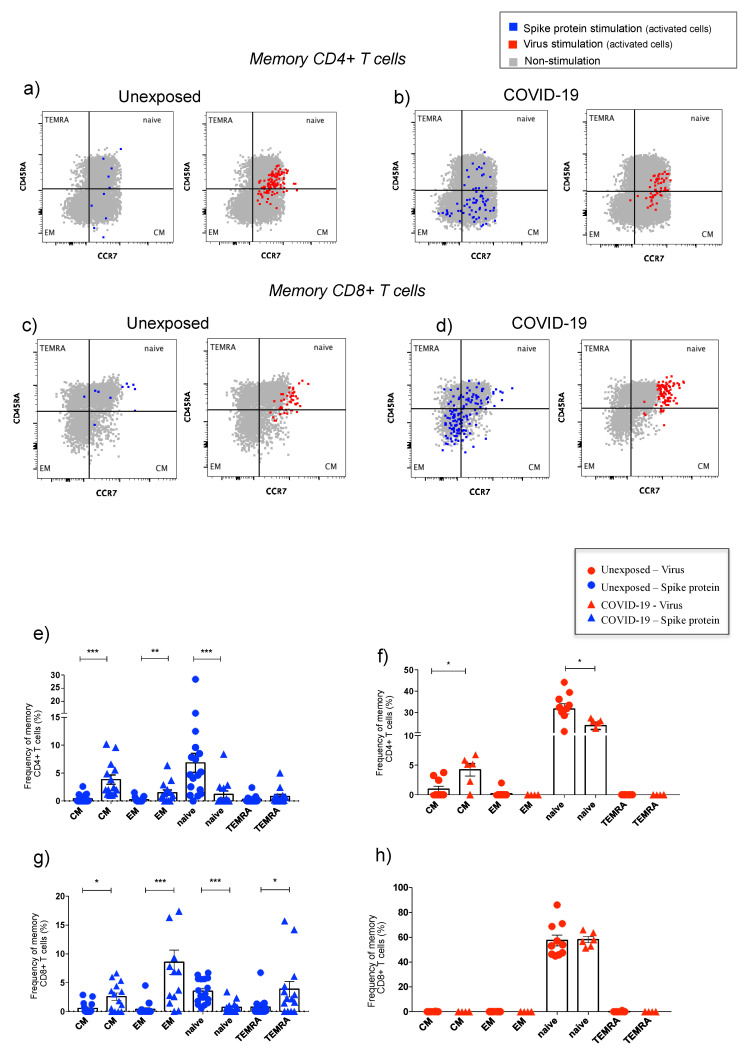
Memory T-cells phenotypes affected by spike protein stimulation and by live SARS-CoV-2 exposure. Percentages of memory T-cells phenotypes, as central memory (CM: CD45RA^−^CCR7^+^), effector memory (EM: CD45RA^−^CCR7^−^), naïve cells (naïve: CD45RA^+^CCR7^+^), and terminally differentiated (TEMRA: CD45RA^+^CCR7^−^) are represented (**a**–**h**). Total of memory T cells are in grey color (bulk: without antigen stimulation), and activated phenotype cells (CD69^+^HLADR^+^) are represented in blue for spike protein stimulation and in red for live virus in dot plot graphs (**a**–**d**). The memory CD4^+^ T (**e**,**f**) and CD8^+^ T cells (**g**,**h**) percentages from unexposed and COVID-19 are displayed by bar graphs. The final percentage value, represented by *y* axis, is the percentage of cells activated by SARS-CoV-2 antigens subtracted from unstimulated cells (% of cells stimulated with spike protein or virus minus % of the cells from mock condition). The data are representative of 2–5 different experiments. Symbols legend represents each sample collected from each volunteer. * *p* < 0.05, ** *p* < 0.01, and *** *p* < 0.001.

Upon spike protein stimulation or SARS-CoV-2-infection, CD4^+^ T and CD8^+^ T cells showed particular features considering the nature of exposition while unexposed, and COVID-19 could represent the first and second virus exposure, respectively, using the in vitro assay (Figure 3, Figure 4 and Figure 5).

CD4^+^ T cell percentage was ~10-fold low compared to CD8+ T cells based on the evaluation of the early activation marker, CD69, together with HLA-DR (marker of cellular activation) after viral infection in both groups of individuals (Figure 3a–c and Figure 4c,d). Spike protein from SARS-CoV-2 showed to be a good T-cell stimulation for COVID-19 (Figure 3b,c and Figure 4c,d,f), with high production of IFN-γ, IL-2, and TNF by CD4^+^ T cells, characterizing the predominant response of Th1 cells. In the COVID-19, Th1 cells were significantly higher than Th2 CD4^+^ T cells, which produced IL-4, IL-2, and TNF, main markers for Th2 response (Figure 3d).

Spike-stimulated PBMCs exhibited high CD8^+^ T-cell frequencies, with IFN-γ, IL-2, and TNF production in COVID-19 (Figure 3d). By contrast, SARS-CoV-2-infected PBMCs showed lower levels of activated CD8^+^ T cells in COVID-19 compared to the unexposed group (Figure 4d).

The apoptotic marker, CD95, was elevated independently of previous disease in CD4^+^ T and CD8^+^ T cells after SARS-CoV-2 infection (Figure 3e,f). However, apoptotic CD8^+^ T cells (CD95^+^) were significantly higher in COVID-19 than unexposed individuals (Figure 3e,f and Figure 4e,f). Meanwhile, spike protein showed no changes for apoptotic T cells (Figure 3e,f and Figure 4e,f).

#### 3.2.4. Memory T Lymphocytes

Memory phenotype of T cells was explored to explain differences among naive and primed T cells. Figure 5 displays analysis of memory phenotypes as central memory (CM: CD45RA^−^CCR7^+^), effector memory (EM: CD45RA^−^CCR7^−^), naïve cells (naïve: CD45RA^+^CCR7^+^), and terminally differentiated (TEMRA: CD45RA^+^CCR7^−^). We observed that unexposed individuals were predominantly naïve either for virus or for spike protein as expected, therefore validating our model (Figure 5).

After spike stimulation, a significant elevation for central and effector memory CD4^+^ T-cells frequencies was detected for COVID-19, followed by all memory subsets for CD8^+^ T cells, except for naive subset, compared to the unexposed group (Figure 5a–e,g). After SARS-CoV-2 infection, the only alteration on the memory T cells was on the frequency of central memory CD4^+^ T cells, also higher in the COVID-19 as compared to the unexposed group (Figure 5f,h).

#### 3.2.5. Expression of SARS-CoV-2 Response Genes

**Figure 6:**
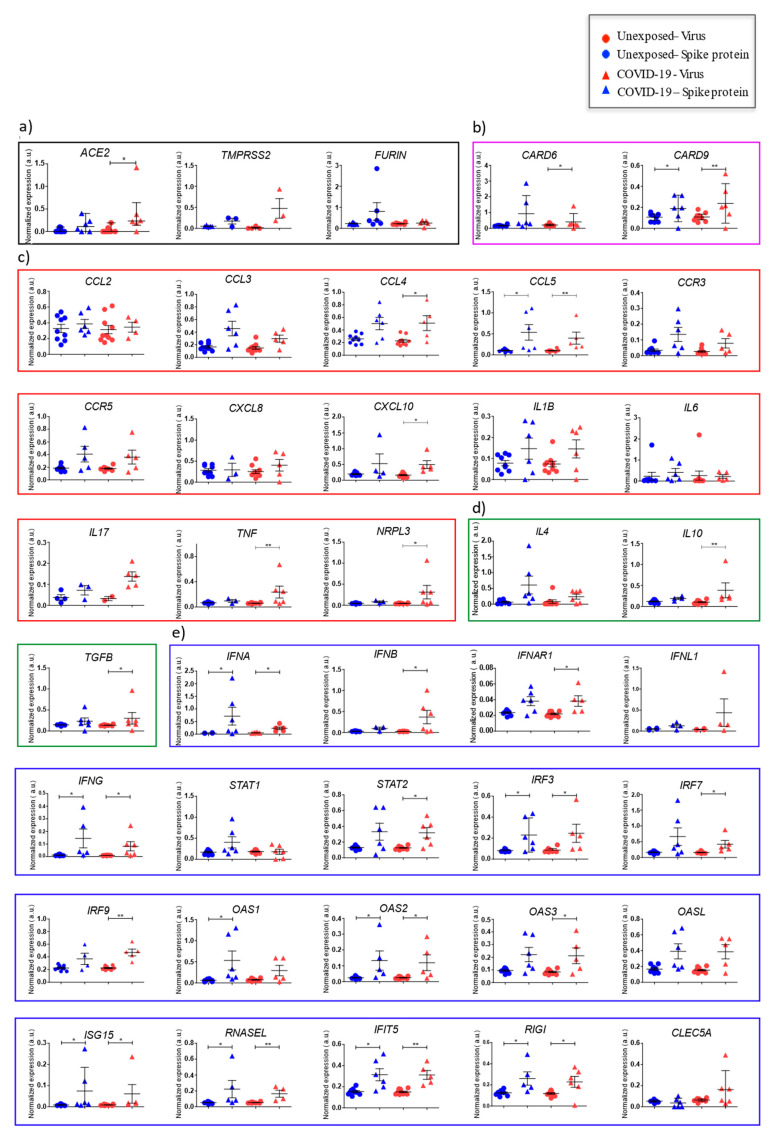
Normalized gene expression of peripheral blood mononuclear subset cells from individuals exposed to SARS-CoV-2 or otherwise after stimuli with spike SARS-CoV-2 protein. Detailed scatted dot plots show normalized expression for genes analyzed in COVID-19 (triangular shapes) or unexposed individuals (circular shape), according to experimental stimuli with spike protein (blue dots) or SARS-CoV-2 (red dots). The number of dots varies according to gene analyzed due to failed amplifications. Boxes represent the median and vertical bars as well as the standard error of normalized expression for each experimental group analyzed using references genes RPL13 and 18S. Genes were clustered in boxes according to biological/immunological functions: SARS-CoV-2 receptors (black) (**a**), apoptosis (purple) (**b**), inflammation (red) (**c**), regulation of inflammation (green) (**d**), and innate antiviral response (blue) (**e**). Graphics and statistical comparisons of normalized expression for unexposed and COVID-19 using Mann–Whitney test in software GraphPad Prism 8.4.2. * *p* < 0.05, ** *p* < 0.01, and *** *p* < 0.001.

Subsequently, we evaluated gene expression patterns associated with biological processes such as virus entry, apoptosis, inflammation, and general innate antiviral response. A slight increase in the frequency of innate cells in the unexposed group was detected. SARS-CoV-2-infected PBMC from COVID-19 presented a significantly higher expression of genes related to virus entry (cellular SARS-CoV-2 receptor *ACE2*) and apoptosis (*CARD6*, and *CARD9*); reinforcing CD8^+^ T cells CD95^+^ immunophenotyping (Figure 6a,b). In addition, our data showed that second exposure to the virus leads to a more intense inflammatory process with higher levels of proinflammatory *CCL4*, *CCL5*, *CXCL10*, *TNF*, and *NRPL3*, together with regulators *IL10* and *TGFB* (Figure 6c,d). Moreover, COVID-19 presented a robust antiviral response including higher levels of *IFNG*, a hallmark of CD8^+^ T cells activity and CD4^+^ T cells Th1 response, type I interferons (*IFNA* and *IFNB*) and their receptors (*IFNAR1*), transcriptional regulators (*STAT2*, *IRF3*, *IRF7*, and *IRF9*) and interferon-stimulated genes (*OAS2*, *OAS3*, *ISG15*, *RNASEL*, *IFIT5*, and *RIGI*) (Figure 6e).

Spike COVID-19 protein stimulation also leads to increased gene expression of *CARD9*, and *CCL5* (Figure 6b,c). A remarkable increase for genes related to interferon response was observed (*IFNA*, *IFNG*, *IRF3*, *OAS1*, *OAS2*, *ISG15*, *RNASEL*, *IFIT5*, and *RIGI*), in contrast with the unexposed group (Figure 6e).

### 3.3. Step2: Effects of Supernatant from PBMC Cell Cultivation in Lung Alveolar Epithelial Cells

The supernatant from SARS-CoV-2-infected PBMCs was used as a conditioned medium to evaluate the interaction among soluble mediators of immune response and lung alveolar epithelial cells (A549). The replication of SARS-CoV-2 on A549 cells was measured by dsRNA staining, in which infected A549 cells expressed dsRNA, as expected (Table 4). A slight decrease in dsRNA expression was observed comparing A549 infected and A549 noninfected cells in the presence of supernatant derived from PBMC virus exposition for both groups, unexposed and COVID-19, but not significant (Table 4).

**Figure 7:**
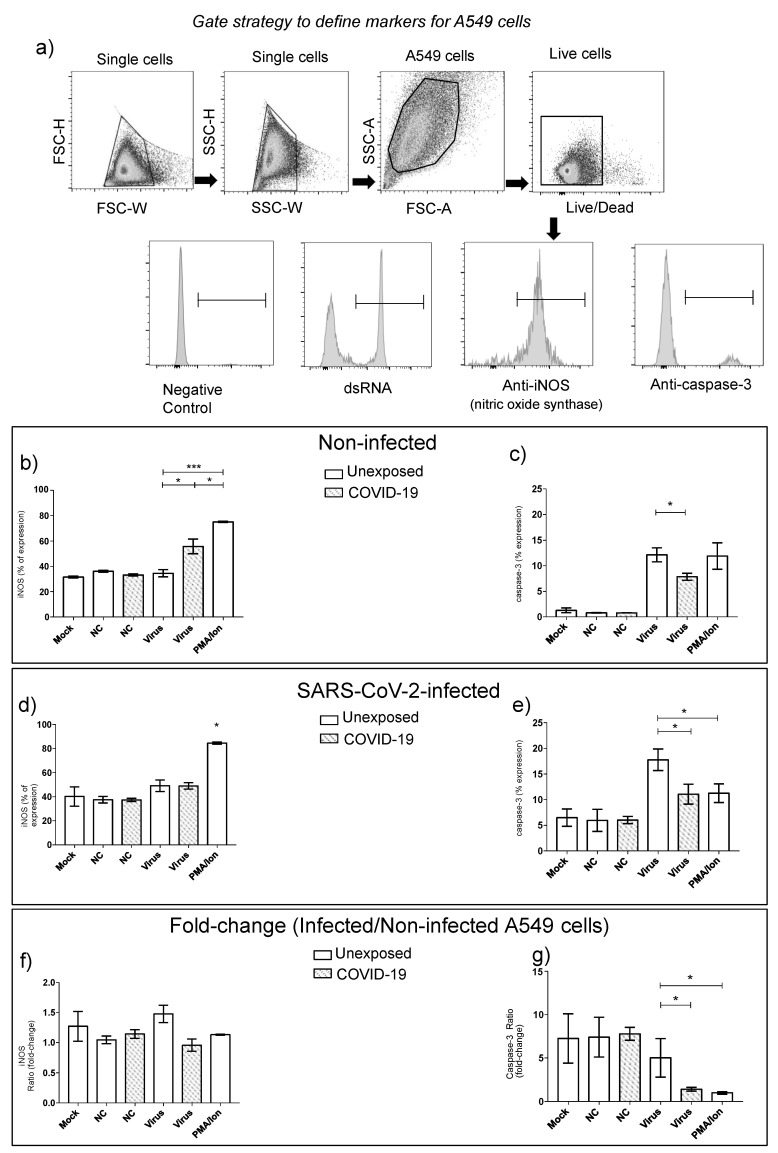
Effects of supernatant from PBMC cell cultivation on lung alveolar epithelial cells. A549 cell data of nitric oxide synthase (iNOS), double-strand RNA (dsRNA), and caspase-3 expression evaluation (**a**). Data from the supernatant from unexposed and COVID-19 PBMC cultivation with live SARS-CoV-2 expressing iNOS (**b**) and caspase-3 (**c**) in A549 noninfected cells. Data from the A549 cells expressing iNOS (**d**) and caspase-3 (**e**) after 48 h of SARS-CoV-2 infection. The fold-change (infected/noninfected) of iNOS (**f**) and caspase-3 (**g**) expression by A549 cells. The supernatant pool from unexposed and COVID-19 PBMC on incubation with SARS-CoV-2 was used. PMA/Ion: Supernatant from proliferative T-cell responses stimulated by polyclonal activator. NC- supernatant from negative control used in the antigen-stimulation assay. The data are representative of 2–5 different experiments. * *p* < 0.05, ** *p* < 0.01, and *** *p* < 0.001.

The antiviral defense activation evaluation by nitric oxide synthase expression (iNOS), as well as the apoptotic effect (caspase-3 expression) of PBMCs supernatants in A549 cells, infected or not, was performed (Figure 7). Cellular responses from unexposed individuals did not change the iNOS expression by A549 (Figure 7b,f), even after SARS-CoV-2 infection of the alveolar epithelial cells (Figure 7d,f). On the other hand, the caspase-3 expressed by A549 frequencies was elevated after PBMC supernatant from unexposed subjects compared to COVID-19 (Figure 7c,e).

The contact with the live virus by PBMC from primo-infected subjects displayed a significant elevation of A549 expressing iNOS, as well as a reduction of caspase-3 by those cells before SARS-CoV-2 infection compared to the unexposed group (Figure 7b,c). After viral infection of alveolar epithelial cells, the product of cellular immune responses from COVID-19 remained inducing the reduction of caspase-3 expression by A549 cells (Figure 7e,g).

## 4. Discussion

Several studies have already demonstrated that the new coronavirus (SARS-CoV-2) is able to infect not only cells in the respiratory tract, but other organs, such as blood [30,31,32,33]. Our findings showed that peripheral blood mononuclear cells, such as B and T lymphocytes, monocytes, and natural killer cells, can be infected by SARS-CoV-2. Pontelli et al. (2020) [13] found the SARS-CoV-2 replication on the same cells explored here, but not for natural killer cells subsets. Meanwhile, some studies supported the decrease in NK cells frequencies in peripheral blood [34], and their inflammatory function on the pulmonary tract in post-mortem COVID-19 cases [35]. Intriguingly, our results for the natural killer cells displayed a high percentage of cytotoxic phenotype (NK^dim^) in unexposed individuals, thus corroborating their activation by viral proteins similarly observed at NK derived from peripheral blood of COVID-19 patients [36].

Monocytes percentages were reduced after in vitro virus exposure and in severe COVID-19 cases, as described by others [37,38]. Nevertheless, a slight increase in nonclassical monocytes (CD14^−^CD16^+^) was observed in the unexposed group after SARS-CoV-2 contact, which may be induced by viral proteins, as noted in the blood of patients with moderate COVID-19 compared to controls (unexposed subjects) [37].

Natural killer cells are the main cell type related to proinflammatory cytokines production at the first line of immune defense against viruses, and it seems to be involved with monocytes/macrophages in the cytokine storm observed in COVID-19 patients [10,34]. Cytokines produced in this context by NKs and monocytes might be the key in severity, while inhibition of TNF, CXCL8, and IL6 have been identified as possible therapeutic targets [39]. In this regard, our results demonstrated a considerable enhancement of inflammatory mediators in SARS-CoV-2-infected PBMCs from unexposed volunteers related to monocytes and NK cells activation. The global decrease in monocyte percentages observed in our model suggests a nonsustained inflammatory response [40].

Lymphocyte T percentages have been measured as an indicator of severity for COVID-19 outcomes, such as lymphopenia; exhaustion and apoptosis of T cells were noted in COVID-19 patients [41,42,43,44,45,46]. We observed here that all participants showed a high expression of the apoptotic marker CD95 after virus infection (Fas), detected mainly in CD8^+^ T cells. Moreover, there is a consistent correlation between lymphopenia and prognosis [41,42,43,44,45,46]. In addition, histopathological analysis of lungs of post-mortem patients showed an important infiltration by T lymphocytes [47,48].

SARS-CoV-2 proteins have been associated with T-cell activation and memory phenotypes after COVID-19 recovery [46,49,50,51,52,53]. B- and T-cell responses are detected in the blood around 1 week after the onset of symptoms. Regarding the general cell response, we present the following findings: autopsies of patients with COVID-19 revealed increased cell exhaustion and SARS-CoV-2 CD4^+^ expressing IFN-γ, TNF-alpha, and IL-2 indicating a Th1 cellular response [54]. After two weeks of symptoms, cellular phenotypes that have specific memory T cells for SARS-CoV-2 in peripheral blood begin to appear. This process can provide useful information on cellular protective/lasting immunity [55].

Considering the previous exposure to SARS-CoV-2, our data demonstrated that T cells from unexposed individuals are not able to recognize the spike protein, but the virus infection was capable of inducing a robust T cell activation in unexposed similar to the clinical findings [46,49,50,51,52,53].

CD4^+^ T helper has been implicated in the pathogenesis that leads to the inflammation process, as well as being affected by SARS-CoV-2 replication, diminishing the T follicular helper cells, contributing to the loss of antibodies, and reduction of IFN-γ production in COVID-19 patients [35,46,49,50,51,52,53,56]. Our findings showed that spike viral protein led to the activation of CD8^+^ T and CD4^+^ T helper cells producing IFN-γ (Th1), and expression of genes related to interferon pathways in COVID-19 individuals.

Additionally, ex vivo studies with COVID-19 patients showed that reactive CD4^+^ T cells are able to produce high levels of chemokines [49,57,58,59]. Here, we also demonstrated that PBMC from primo-infected individuals after virus infection presented a higher antiviral response including high expression of *IFNG*, a key to Th1 differentiation [60] and CD8^+^ T cell activation [61]. Taken together, these results also suggest that CD4^+^ T cells from COVID-19 could be key players on inflammatory and antiviral responses upon a second exposition, which would be advantageous for the vaccines.

Concerning the memory T cell phenotypes, our results showed that unexposed individuals only have naïve T cells raised after antigen stimulation, as described for viral infections or vaccination [62,63,64] and differently as noted by others [52,53]. Our data corroborate the findings described by Grifoni et al. (2020) [53] about central memory CD4^+^ T-cell activation in subjects recovered from mild COVID-19 infection after stimuli with viral proteins. Moreover, this scenario is expected as a good signal of positive response for vaccines, in which the next contact with a viral protein, or whole virus, could promote T-cell recognition, activation, and consequently full protection with *IFNG* gene expression and IFN-γ production by CD4^+^ T cells [53,55,65].

In the pathway to investigate the output of cellular responses on the alveolar epithelial lung cells (A549) after in vitro exposure to SARS-CoV-2 antigens, the nitric oxide synthase (iNOS) and caspase-3 expression by A549 were assessed aiming at evaluating if their expression can be regulated by cytokines among other mediators produced by infected cells [66,67,68,69,70]. The antiviral activity in supernatants provided alterations in the alveolar epithelial lung cells (A549) during our in vitro assay. Despite no changes of viral replication (dsRNA), the consequences were the reduction of caspase-3 expression in SARS-CoV-2 infected cells and elevation of iNOS expression in noninfected A549 cells. Probably, the cellular immune response may help towards the antiviral activity and could raise the nitric oxide (NO) production by A549 cells, a product of iNOS function, described to be effective in reducing SARS-CoV-2 replication in patients with COVID-19 [71,72]. However, further investigations are needed to better understand the role and complexity of the immune response after SARS-CoV-2 exposure, even for COVID-19.

It is worth noting that our data need to be interpreted in the context of some limitations, as the in vitro assays cannot be compared with clinical and physiological data. On the other hand, in vitro models are the first step to understand the virus–host interactions bringing very useful information to minimize exposure to the virus and the need for assays to be carried out in biosafety laboratories level III. Adding to that, we did not explore the possibility of the antibody dependent enhancement, whereas on the second exposure the major immune response for CD4^+^ Th1 cells was a good trigger for further investigations.

The use of conditioned medium (CM) obtained from the supernatant derived from cell cultivation has been used to study the effects of those components on in vitro assays to investigate their effects on different cell types [73,74]. However, there is no information about the use of supernatant from PBMC cultivation after SARS-CoV-2 infection/stimulation on alveolar epithelial cells or other cell lines.

In sum, our data brought a new way to explore the effects of SARS-CoV-2 infection on the cellular immune response using a two-step in vitro model, showing the differences between naive response and SARS-CoV-2 primed response. In our Step1 assay, the results showed that natural killer cells played an important role in the immune response for the unexposed individuals and may induce the immune imbalance in COVID-19. CD4^+^ T lymphocytes producing IFN-γ were the principal phenotype for memory T cells after virus exposure in COVID-19 and can be the key for vaccine development and protective evaluation, as well as the *IFNG* expression with antiviral genes. In our Step2 assay, we observed positive effects on apoptosis reduction in alveolar cells infected by the new coronavirus using the product of virus-specific immune response by people who were exposed previously to SARS-CoV-2, thus suggesting that a second exposure might collaborate with a strong immune response, mainly for those who showed mild symptoms, without hospitalization. Therefore, considering the emergence of new variants, further studies are necessary to the ongoing comprehension of the SARS-CoV-2 immunity even after infection or vaccination. Our findings reinforce that cellular immune response is key to a better understanding of the host interactions in COVID-19. The two-step in vitro models can help further studies for other immunological assays or might be used as a screening system to test new immunotherapy products.

## Figures and Tables

**Table 1:** Clinical characteristics of the study population.

ID	Gender	RT-PCR SARS-CoV-2	Serology IgM/IgG	Signals & Symptoms	Age	Spike Assay (BSL2)	Virus Assay (BSL3)
1	F	Negative	Negative	No	45	Yes	No
2	M	Positive	Positive	Yes	44	Yes	Yes
3	F	Positive	Negative	Yes	28	Yes	No
4	F	Positive	Negative	Yes	38	Yes	No
5	F	Positive	Negative	Yes	37	Yes	No
6	M	Positive	Negative	Yes	29	Yes	No
7	M	Positive	Positive	Yes	28	Yes	No
8	F	Negative	Negative	No	32	Yes	No
9	F	Positive	Positive	Yes	32	Yes	Yes
10	F	Negative	Negative	No	29	Yes	No
11	M	Negative	Negative	No	31	Yes	No
12	M	Negative	Negative	No	45	Yes	Yes
13	F	Negative	Negative	No	42	Yes	Yes
14	F	Negative	Negative	No	31	Yes	Yes
15	F	Negative	Negative	No	52	Yes	No
16	F	Negative	Negative	No	39	Yes	Yes
17	M	Negative	Negative	No	26	Yes	No
18	M	Negative	Negative	No	24	Yes	No
19	F	Negative	Negative	No	28	Yes	Yes
20	F	Negative	Negative	No	30	Yes	No
21	F	Negative	Negative	No	36	Yes	Yes
22	M	Negative	Negative	No	38	Yes	Yes
23	M	Negative	Negative	No	38	Yes	Yes
24	M	Negative	Negative	No	31	Yes	Yes
25	M	Negative	Negative	No	29	Yes	Yes
26	F	Positive	Positive	Yes	34	Yes	Yes
27	M	Positive	Positive	Yes	56	Yes	Yes
28	F	Positive	Positive	Yes	58	Yes	Yes
29	M	Positive	Positive	Yes	34	Yes	Yes

The signal and symptoms reported by volunteers who had COVID-19 confirmed were fever, headache, myalgia, cough, and loss of smell/taste; each volunteer reported at least three of those symptoms. The duration of symptoms lasted up to two weeks. F: female, M: male. Descriptions about the assays performed in biosafety level II (BSL2) laboratory and biosafety level III (BSL3) laboratory. Blood draw was performed on May 2020.

**Table 2:** Descriptive frequencies of clinical characteristics.

		Unexposed (*n* = 18)	COVID-19 (*n* = 11)
RT-qPCR SARS-CoV-2	Positive	__	11 (100%)
	Negative	18 (100%)	__
Serology IgM/IgG	Positive	__	7 (63.6%)
	Negative	18 (100%)	4 (36.4%)
Signals and symptoms	Yes	__	11 (100%)
	No	18 (100%)	__
Demographic	Gender (F; M)	10; 8	6; 5
	Age (mean ± SD)	35 ± 8	38 ± 11

**Table 3:** Absolute frequency of circulating peripheral blood mononuclear cells from the study population.

	Unexposed (Mean ± SE)	COVID-19 (Mean ± SE)	*p* Value
T cells	36.75 ± 4.03	30.7 ± 3.00	0.48
B cells	15.25 ± 4.75	8.48 ± 3.38	0.22
Monocytes	2.73 ± 1.68	3.31 ± 1.55	0.25
Natural killer	1.61 ± 0.45	1.34 ± 0.27	0.11

Data of the absolute frequency from PBMC are expressed as percentage (%). The identification of the cell phenotypes were according to the gate strategy described in Figure 1a. SE: standard error.

**Table 4:** Frequency of A549 cells (%) expressing dsRNA.

	Infected	Noninfected	*p* Value Infected vs. Noninfected
Mock A549	56.34 ± 3.34	1.47 ± 0.32	0.018
NC (Unexposed)	57.82 ± 1.25	1.48 ± 0.43	0.019
NC (COVID-19)	53.71 ± 8.32	1.40 ± 0.33	0.024
Virus (Unexposed)	54.32 ± 6.43	42.2 ± 7.31	0.221
Virus (COVID-19)	55.61 ± 7.82	42.3 ± 9.60	0.250

Data are represented by mean ± SE. NC: negative control, as supernatant from mock condition of PBMC cultivation.

## Data Availability

Not applicable.
